# Seasonal Dynamics of the Alien Invasive Insect Pest *Spodoptera frugiperda* Smith (Lepidoptera: Noctuidae) in Manica Province, Central Mozambique

**DOI:** 10.3390/insects11080512

**Published:** 2020-08-07

**Authors:** Albasini Caniço, António Mexia, Luisa Santos

**Affiliations:** 1 LEAF-Linking Landscape, Environment, Agriculture and Food- School of Agriculture—University of Lisbon, Tapada da Ajuda, 1349-017 Lisbon, Portugal; amexia@isa.ulisboa.pt; 2 Division of Agriculture—The Polytechnic of Manica (ISPM), District of Vanduzi, Matsinho 2200, Mozambique; 3 Department of Plant Protection—Faculty of Agronomy and Forestry Engineering, Eduardo, Mondlane University, P.O. Box 257, Maputo 1102, Mozambique; luisasantos47@gmail.com

**Keywords:** fall armyworm, cropping season, population density, infestation, smallholder farmers

## Abstract

**Simple Summary:**

In this article “Seasonal dynamics of the alien insect pest *Spodoptera frugiperda* Smith (Lepidoptera: Noctuidae) in Manica province, central Mozambique”, the authors Albasini Caniço, António Mexia and Luisa Santos, discuss the population fluctuation of a newly introduced and important insect pest. This insect pest attacks maize, which is a staple food in the country. Because the pest is highly voracious, its attack on maize reduces the grain yield and threatens food security of around half of the Mozambican population. The authors compared the situation of the pest in the dry and rainy seasons. The study shows that during the dry season, the population of this pest increases and many plants are attacked and eventually killed. With this knowledge, farmers and researchers can efficiently plan about when the control measures should be stepped up to deal with this insect pest.

**Abstract:**

The alien invasive insect pest *Spodoptera frugiperda* Smith (Lepidoptera: Noctuidae), commonly referred to as fall armyworm (FAW), is causing significant losses to maize production in Africa since its detection in 2016. Despite being the primary insect pest of the main food crop in the country, researchers have concentrated their efforts on methods of control, and there are no published studies on its seasonality which could assist farmers in delivering effective methods of control in periods of heavy infestations. The primary goal of this study was to assess the seasonal dynamics of FAW in maize fields. We conducted a field survey from May to August 2019 (dry season of the 2018/2019 cropping season) and in December 2019 and January 2020 (rainy season of the 2019/2020 cropping season) in 622 maize fields. In each field, 20 plants were selected in a “W” pattern and checked for the presence of FAW egg masses and/or larvae. Plants were also assessed for damage. Preliminary results show increased infestation, damages, and population density of FAW in the dry season. Our results suggest that early planting of maize in the primary cropping season may significantly reduce the infestation and damage by FAW when compared to the dry season.

## 1. Introduction

The fall armyworm *Spodoptera frugiperda* Smith (Lepidoptera: Noctuidae) is an alien polyphagous insect pest originating from the Americas, where it has more than 350 different host plants including both crop and non-crop species [1]. Despite its ability to survive in different host plants, fall armyworm (FAW) is known to have a high preference for maize [2,3]. In Africa, FAW was first reported in West and Central Africa in 2016 [4] and rapidly spread to the rest of the continent with devastating consequences on maize production [5]. Initially confused with stem borers by agricultural extension officers, the occurrence of FAW in Mozambique was confirmed in early 2017 by the Ministry of Agriculture and Food Security [6]. In 2018, FAW was also reported in Asia [7]. The rapid spread of FAW is attributed mainly to its migratory potential [8] and high dispersal capacity [9].

Alien invasive species are known to disrupt the natural balance in newly invaded ecosystems, creating severe problems [10]. This is the case of FAW, which threatens food security in Sub-Saharan Africa where maize is a staple food [11,12,13,14]. In Mozambique, for example, the percentage of households which depends on maize for daily subsistence ranges from 21 to 90%, with a national average of around 44% [15].

In Mozambique, maize is cultivated in both dry and rainy seasons. The rainy season starts from mid-November to late March. During the dry season, maize is cultivated mainly in areas with irrigation systems or in valleys and riverbanks. It is often grown in small plots (less than 1 ha) under different cropping systems and mainly for family consumption. In general, no fertilizers and chemicals are used for maize production at smallholder farmers’ level. It is usually intercropped with roots and tubers, legumes, and cucurbits.

Similarly to other insect pests, FAW is known to be affected by weather conditions of different seasons. The number of FAW individuals in a given area is believed to be directly influenced, among other factors, by the time of the year, weather conditions, and availability of host plants [16]. In its native habitat, for example, FAW can be found in maize fields in all cropping seasons [17]. But in other places, such as the southeast region of the United States, FAW is considered a sporadic pest due to weather conditions of those regions which are not suitable in some periods of the year [18]. When weather conditions are not favorable for its development and reproduction, FAW is forced to migrate to more suitable locations for its survival [19,20].

Being originally a tropical insect [19], FAW performs better in hot climates [21,22]. The lower and upper limits of tolerance of temperature are 10 [23] and 42 °C [24], respectively. The optimal range of temperature for its development is between 30 and 35 °C, and its survival and development rates do not seem to be affected by humidity [23]. Depending on the temperature, the development cycle of FAW can be significantly affected [25].

In Sub-Saharan Africa, where the temperatures are similar to those of its native area, it is believed that FAW also occurs all year long [26]. A study on the seasonality of FAW in Northern Ghana [27] suggested that the abundance of the pest was influenced by temperature, rain, and relative humidity of different seasons. In Mozambique, where FAW is a new insect pest, there are no published studies of its seasonality which could assist smallholder farmers in concentrating and probably coordinate control options in periods of higher infestations and damages. In this study, we aimed to assess the seasonal dynamics of FAW in maize fields in the central province of Manica, Mozambique.

## 2. Materials and Methods

### 2.1. Description of the Study Area

**Figure 1:**
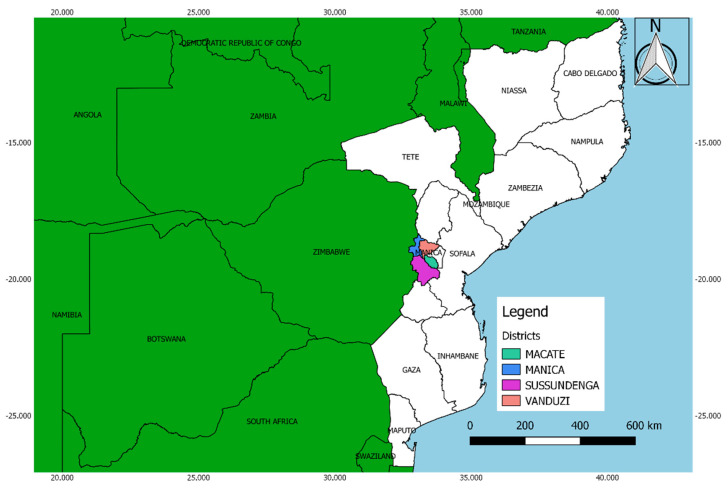
Sampling locations in Mozambique.

This study was carried out in the districts of Macate (19°24′50.9″ south and 33°30′54.6″ east), Manica (18°56′13.2″ south and 32°52′33.6″ east), Sussundenga (19°24′39.0″ south and 33°16′33.0″ east), and Vanduzi (18°57′09.4″ south and 33°15′51.6″ east) in the central province of Manica, Mozambique (Figure 1). According to [15], the area of the survey belongs to the agro-ecological region (AER) number 4, which is characterized by the extensive occurrence of ferralsols and lithosols with an annual mean temperature around 24 °C and annual mean precipitation ranging between 800 and 1000 mm.

### 2.2. Survey of Fall Armyworm

Surveys were carried out from May to August 2019 (dry season of 2018/2019 cropping season) and in December 2019 and January 2020 (rainy season of 2019/2020 cropping season). Districts were selected based on their potential for maize production combined with the reported occurrence of FAW. A total of 622 fields were surveyed in dry and rainy seasons including 25 and 131 in Macate, 29 and 137 in Manica, 27 and 141 in Sussundenga, and 59 and 73 in Vanduzi, respectively. Districts were visited once per month. Each field was visited once during the study period. Fields were selected using a snowball sampling technique. Only fields with at least 200 plants were selected. Based on the illustration of maize growth stages by [28], only fields in which plants were in stages 1 to 5 were sampled. To avoid border effects, in fields in which maize was planted in rows, the first two border rows were excluded from the survey. In fields in which maize was not planted in rows, an estimated distance of 1 m from the border was excluded from the survey on either side of the field. In each field, 20 plants were selected in a “W” pattern and checked for the presence of FAW egg masses and/or larvae. A distance of 3m between plants was observed. Stalks and both upper and lower surfaces of plant leaves were inspected. The number of egg masses and larvae present in each plant was recorded. The number of infested plants and plants damaged as a consequence of FAW attack was also recorded. Foliar damage was assessed based on a visual scale ranging from 0 to 5 scores as described: 0 = plant with no visual foliar damage; 1 = up to 10% of foliar damage; 2 = foliar damage between 10 to 25%; 3 = foliar damage between 25 to 50%; 4 = foliar damage between 50 to 75%; 5 = more than 75% of foliar damage or a dead plant due to FAW attack. Field surveys were carried out during the daylight period, from 7 h to 17 h, and no trap was used to monitor adult moths. Given that the pupal stage of FAW normally occurs in the soil, this stage was deliberately excluded from the survey. In very few cases which came to our attention, sprayed fields were also excluded from the survey.

### 2.3. Variables

#### 2.3.1. Percentage of Infested Fields

The percentage of infested fields per district (FI) was determined by dividing the number of fields in which FAW egg masses and/or larvae were recorded (Fi) by the total number of fields surveyed (Ft) and converted to per cent values (Equation (1)). Fields were considered as being infested whenever at least 1 out of 20 plants observed per field contained FAW egg masses and/or larvae.

(1) $$\mathrm{FI}=\frac{\mathrm{Fi}}{\mathrm{Ft}}\;*\;100\%$$

#### 2.3.2. Percentage of Infested Plants

The percentage of infested plants per field (PI) was determined by dividing the number of plants found to contain FAW egg masses and/or larvae (Pi) by the total number of plants surveyed (Pt) and converted to per cent values (Equation (2)). Plants were considered as being infested whenever FAW egg masses and/or larvae were recorded.

(2) $$\mathrm{PI}=\frac{\mathrm{Pi}}{\mathrm{Pt}}\;*\;100\%$$

#### 2.3.3. Percentage of Damaged Plants

The percentage of damaged plants per field (PD) was determined by dividing the number of plants with visual symptoms of FAW attack (Pd) by the total number of plants surveyed (Pt) and converted to per cent values (Equation (3)). Plants were considered as being damaged every time visual symptoms of FAW attack were recorded, regardless of the presence or absence of feeding larvae.

(3) $$\mathrm{PD}=\frac{\mathrm{Pd}}{\mathrm{Pt}}\;*\;100\%$$

#### 2.3.4. Average Plant Damage

The average plant damage per field (LD) was determined by dividing the sum of scores of individual plants (∑Di) by the total number of plants surveyed (Pt) (Equation (4)).

(4) $$\mathrm{LD}=\frac{\sum \mathrm{Di}}{\mathrm{Pt}}$$

#### 2.3.5. Number of FAW Egg Masses per Field

The average number of FAW egg masses per field (EG) was determined by dividing the number of recorded egg masses per district (Er) by the total number of fields surveyed in the district (Fd) (Equation (5)).

(5) $$\mathrm{EG}=\frac{\mathrm{Er}}{\mathrm{Fd}}$$

#### 2.3.6. Number of FAW Larvae per Field

The average number of FAW larvae per field (LD) was determined by dividing the number of larvae recorded per district (Lr) by the total number of fields surveyed in the district (Fd) (Equation (6)).

(6) $$\mathrm{LD}\;=\frac{\mathrm{Lr}}{\mathrm{Fd}}$$

### 2.4. Meteorological Data

Monthly mean temperatures and precipitation of the study period were obtained from the office of the National Institute of Meteorology (INAM) in Manica province, which is responsible for monitoring the weather in the study area. Due to the unavailability of meteorological data from the districts of Vanduzi and Macate, we used data from the closest weather stations of Chimoio and Gondola, respectively.

### 2.5. Data Analysis

Data analysis was performed through R Statistical Software version 3.6.1 (Action of the Toes). Mean differences of the percentage of damaged and infested plants and the average number of egg masses and larvae per field between seasons in the same district were assessed through a *t*-test at 95% confidence interval (File S2). One-way analysis of variance (α = 0.05) was performed to detect differences on the percentage of damaged and infested plants and the average number of egg masses and larvae per field among districts within the same season of sampling (File S1). Mean separation on these variables was performed through a Tukey honestly significant difference test (Tukey HSD) at 95% family-wise confidence level. Differences in damage scores per field within the same district in different seasons, and among districts in the same season, were assessed based on the points of the scale used.

## 3. Results

### 3.1. Infestation

Table 1 (below) shows the percentage of infested fields and infested plants per field per district and season of sampling. In the dry season, the percentage of infested fields ranged from 60 to 82.76%, while in the rainy season, the values ranged from 14.18 to 34.25%. The percentage of infested plants per field was higher in the districts of Sussundenga and Manica (*p* = 0.008), although Manica did not differ from Macate and Vanduzi. For the rainy season, a higher percentage of infested plants was recorded in the district of Vanduzi (*p* < 0.001). When comparisons were made between seasons, the percentage of infested plants per field was higher in the dry season in all districts.

### 3.2. Damage

Table 2 shows the percentage of damaged plants per field and average plant damage scores per field per district and season of sampling. No differences were observed in the percentage of damaged plants per field among districts in the dry season (*p* = 0.117) but, in the rainy season, the district of Sussundenga exhibited a lower percentage of damaged plants per field (*p* = 0.004), which in turn was not different from Macate and Manica. Between seasons, the percentage of damaged plants per field was higher in the dry season than in the rainy season in all districts.

In the dry season, the average plant damage was more intense in the district of Sussundenga 3 scores, which means that between 25 and 50% of the plant surface appeared to be damaged by FAW larvae. Still, no differences were observed on damage intensity in the rainy season among districts. When damage intensity was compared within the same district between seasons, dry season once again showed higher values than those recorded in the rainy season.

### 3.3. Number of FAW Egg Masses and Larvae per Field

Table 3 shows the average number of FAW egg masses and larvae per field per district and season of sampling. No differences were observed in the number of FAW egg masses per field within the same season among districts, nor between seasons in the same district. While the number of FAW larvae per field was higher in the district of Sussundenga during the dry season (*p* < 0.001), in the rainy season, the district of Vanduzi was the one with higher values (*p* < 0.001). Between seasons, all districts had a higher number of larvae per field in the dry season.

### 3.4. Temperature and Precipitation during the Survey

**Figure 2:**
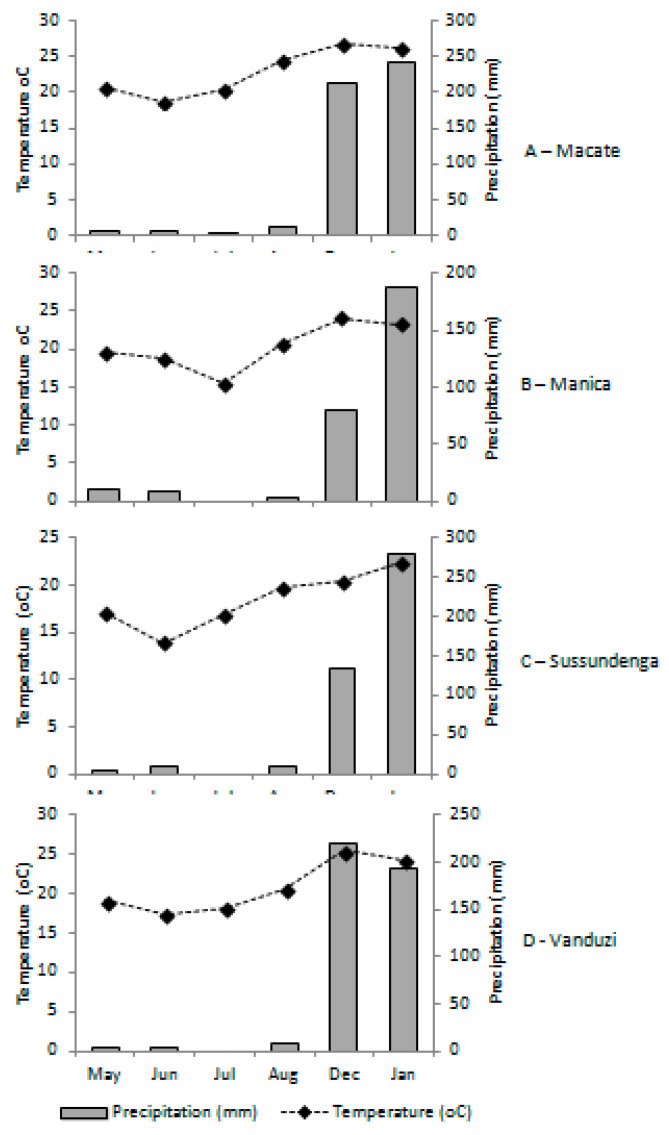
Monthly mean temperatures (°C) and mean precipitation (mm) in the districts of Macate, Manica, Sussundenga, and Vanduzi in the dry season (May to August) and in the rainy season (December and January).

An increase in the average monthly temperatures can be observed during the rainy season when compared with the dry season. A similar pattern was also observed in the case of rain, where huge differences were recorded between seasons (Figure 2).

In Macate, the temperatures of the dry season varied from 18.7 to 24.4 °C, while in the rainy season ranged from 26.3 to 26.9 °C. While the precipitation varied from 1.2 to 10.9 mm in the dry season, in the rainy season, it varied from 212.2 to 241.8 mm. In Manica, the temperatures ranged from 15.4 to 20.7 °C during the dry season and from 23.3 to 23.9 °C in the rainy season. However, the precipitation varied from 0 to 10.9 mm during the dry season and from 80.5 to 186.8 mm during the rainy season. In Sussundenga, the temperatures of the dry season ranged from 13.9 to 19.7 °C. In contrast, for the rainy season, the temperatures varied from 20.5 to 22.5 °C. The precipitation for Sussundenga ranged from 0 to 8.7 mm in the dry season and from 134.9 to 279.4 mm in the rainy season. In Vanduzi, the temperatures of the dry season varied from 17.4 to 20.5 °C, while in the rainy season varied from 24.2 to 25.5 °C. The precipitation of the dry season varied from 0 to 8.2 mm, while that of the rainy season varied from 193.2 to 220.6 mm.

## 4. Discussion

In our study, the number of infested plants per field (Table 1) was lower than the number of damaged plants (Table 2). This result was likely due to the short period of larval development when compared to the length of the period of maize vegetative stage, as larvae might have reached the adult stage and abandoned damaged plants. Some plants which were found to be damaged were not necessarily infested at the time of the sampling.

Although we did not record the growth stages of maize plants in each field, growth stages at the time of the sampling might have played a role in the levels of infestation and damages observed among districts and between seasons. In their study, [29] found that at the plant level, the infestation by FAW was age-dependent because younger stages of maize were found to be more infested than older stages. The sampling interval observed during this study might also have affected the results as conditions varied in different months.

We expected to record higher numbers of FAW egg masses and larvae during the rainy season due to more availability of food in this period compared to the dry season, which would result in more significant foliar damages and infestation. However, we observed a contrary tendency as the number of egg masses and larvae recorded in the rainy season were much lower than those found on the dry season, although the number of maize fields sampled in the rainy season was by far higher than during the dry season.

There was a slight difference in temperatures between seasons (Figure 2). Unlike temperature, the difference in rainfall between seasons was noticeably big. Our results suggest that rainfall was a key factor influencing the differences observed in the number of FAW egg masses and larvae per field between seasons in all districts and that temperature did not affect the survival of FAW.

Climatic factors are believed to directly affect the survival and abundance of pest species [30] as was observed in Nicaragua [31] when they recorded an increase of FAW population during the dry season. Precipitation is another critical factor which has a direct negative effect on larval and pupal survival of FAW [26].

Concerning the rain, several studies [25,26] suggested that the population density of FAW is negatively influenced by pluviometric conditions because when the maize whorl is filled up with water, the larvae of FAW are forced to abandon the whorl. In contrast, egg masses and small larvae are washed off onto the ground, reducing, by consequence, the pest population. Our results on FAW population during the rainy season seem to follow the hypothesis of reduction of its population as a consequence of the rainy weather which occurs from mid-November to late March as it might have significantly affected the survival rate of FAW. Our findings suggest that the dynamics of FAW seems to be more influenced by the prevailing climatic conditions rather than by the number of maize fields available.

Among several weather factors, temperature plays a key role in the survival and development of FAW [27,28]. Studying the seasonality of FAW and other noctuid species, it was observed that an increase in the temperature resulted in the build-up of its population [22]. Our results suggest that the potential effects of temperature on FAW population are nullified by the amount of rain occurring in the same period, as rain has adverse impacts on FAW population. The differences in the mean temperatures between seasons are not significant enough to create specific conditions which could have influenced FAW population differently, as in both seasons, the mean temperatures are situated within the favorable range for its development.

In East Africa (Kenya, Tanzania, and Uganda), a close relative of FAW, the noctuid *Spodoptera exempta* Walker, seems to exhibit a contrasting behavior as its peak occurs between December and May [24]. However, the weather conditions are not very different from those of Mozambique in the same period. In their study of seasonal abundance of FAW in Florida, USA [32], they recorded very low numbers of moths between December and April in two consecutive years. Studying the seasonal distribution of FAW in southern Florida [33], they concluded that the reduction of the amount of rain had a positive effect on the population of FAW. Although Florida is in the northern hemisphere, its rainy season occurs in the same period as in the southern hemisphere where Mozambique is located. Therefore, the hypothesis that rain affects FAW abundance might explain the numbers of FAW recorded in both seasons of our study.

Another important factor affecting FAW dynamics in maize fields is altitude. Analyzing the influence of altitude in the abundance of FAW [34], it was concluded that there was a negative correlation between the abundance of FAW and altitude, as fields located in higher altitudes were less infested than those located in lower altitudes. Despite the existence of slight differences in altitude among sampling locations (from 542 m above sea level in Sussundenga to 679 m above sea level in Manica), differences observed might have been caused by factors other than altitude as the sampling locations are considered as being in the same range of altitude.

While different levels of infestation and damage may affect the yield differently [35], in our study, both infestation and damage were higher in the dry season. They might have had a different influence on the yield when compared with the rainy season. Based on the relationship between the percentage of FAW-infested plants and yield on maize [17], the infestation recorded in our study during the dry season might have caused a yield reduction ranging from 11% in the district of Macate to 27% in the district of Sussundenga compared to potential yield reduction ranging from around 2% in the district of Macate to 8% in the district of Vanduzi in the rainy season.

The knowledge of the dynamics of a pest population is a fundamental tool for the implementation of integrated pest management strategies. In temperate climates, where winter temperatures are shallow and not suitable for development and reproduction of FAW, its population is limited to the summer [36]. Monitoring the populations of two Lepidopteran Noctuid species in the United States, it was found that the peak of the populations of both species occurred in the spring [37]. This trend was also confirmed for FAW [38]. It is important to note that during the spring in some regions of the United States, the weather conditions are similar to those of winter (dry season) in tropical countries like Mozambique.

Our results show that the population density of FAW is higher in the dry season than in the rainy season. Nevertheless, [39] reported a contradicting scenario in their study about the infestation of FAW in pasture grasses in French Guiana, where the highest number of FAW larvae was observed during the rainy season and the lowest in the dry season. Another contradicting scenario was also reported in Northern Ghana, where the rainy season positively influenced the population of FAW in maize fields [27]. These conflicting scenarios reinforce the hypothesis that the dynamic of a pest population is a complex issue, given that the pest itself is influenced by climate and weather which in turn are also complex and dynamic [30].

For unknown reasons, FAW is differently affected by rain. While in some places rain has positive effects on FAW population, in other locations the very same element acts in the opposite direction. Although there may exist other factors contributing to the regulation of FAW population which we may not be aware of, the continuous availability of maize throughout the year combined with weather conditions seem to play a more significant role in the dynamics of FAW in Manica province.

Agricultural practices and cropping patterns that may change with the season are believed to influence the evolution and population dynamics of insect pests [40]. However, our results do not fit in this assumption as, traditionally, cropping patterns used by smallholder farmers in Mozambique do not change that much, given that same crops are cultivated in both dry and rainy seasons, varying only in the number of fields per season. Therefore, cropping patterns do not appear to be a determinant factor of FAW dynamics in Manica province.

## 5. Conclusions

Our study shows that FAW occurs in both dry and rainy seasons, but infestation and damage levels are higher in the dry season. Unlike larvae, of which the abundance appeared to be profoundly affected by rain, the abundance of FAW egg masses did not seem to be affected by specific weather conditions of each season. Although the temperature may affect the performance of FAW, the slight variation of temperature between seasons did not have an impact on the population dynamics. Data obtained from this study suggests that early planting of maize in the primary cropping season may significantly reduce the population density of FAW, lowering by consequence the infestation and damage caused by FAW when compared to the dry season.

### Study Limitations

Results from this study should not be taken as conclusive given the limited period in which it was carried out. Although our results are preliminary, they shed light on the field-behavior of FAW in the country, considering its pest status and that FAW is a new pest in Mozambique. Given the complexity of the dynamics of insect pests and to generate detailed information about the seasonality of FAW, future surveys should be carried out across years and include both on-farm and on-station experiments in different AER’s of the country. On-farm and on-station experiments would allow multiple visits to the same fields during the growth cycle of the crop and the gathering of data related to the monthly fluctuation of FAW population throughout the year.

## Figures and Tables

**Table 1:** Percentage of infested fields and average infestation of plants per field, district, and season of sampling.

District	% of Infested Fields		% of Infested Plants Per Field (Mean ± SD)	
	Dry Season	Rainy Season	Dry Season	Rainy Season
Macate	60.00	16.15	31.00 ± (38.94) Ba	2.62 ± (7.02) Bb
Manica	82.76	23.36	48.45 ± (35.36) ABa	5.62 ± (14.49) Bb
Sussundenga	81.48	14.18	66.48 ± (37.95) Aa	3.23 ± (9.64) Bb
Vanduzi	71.19	34.25	42.63 ± (38.43) Ba	11.99 ± (21.03) Ab

SD = Standard Deviation. Means ± (SD) followed by the same capital letter in the column are not statistically different. Means ± (SD) followed by the same small letter between columns are not statistically different.

**Table 2:** Percentage of damaged plants per field and average plant damage score per field per district and season of sampling.

District	% of Damaged Plants Per Field (Mean ± SD)		Plant Damage Score Per Field (Scale 0–5) (Mean ± SD)	
	Dry Season	Rainy Season	Dry Season	Rainy Season
Macate	62.4 ± (40.03) Aa	19.35 ± (38.47) ABb	1.33 ± (1.16) Ba	0.33 ± (0.66) Ab
Manica	79.14 ± (35.71) Aa	18.61 ± (33.40) ABb	1.62 ± (0.95) Ba	0.34 ± (0.63) Ab
Sussundenga	81.48 ± (31.31) Aa	11.88 ± (28.43) Bb	2.88 ± (5.04) Aa	0.25 ± (0.61) Ab
Vanduzi	80.59 ± (30.39) Aa	30.27 ± (42.34) Ab	1.51 ± (0.90) Ba	0.69 ± (1.03) Ab

SD = Standard Deviation. Means ± (SD) followed by the same capital letter in the column are not statistically different. Means ± (SD) followed by the same small letter between columns are not statistically different.

**Table 3:** Average number of fall armyworm (FAW) egg masses and larvae per field per district and season of sampling.

District	Number of Egg Masses (mean ± SD)		Number of Larvae (Mean ± SD)	
	Dry Season	Rainy Season	Dry Season	Rainy Season
Macate	0.16 ± (0.62) Aa	0.03 ± (0.35) Aa	7.92 ± (10.36) Ba	0.52 ± (1.40) Bb
Manica	0.69 ± (1.63) Aa	0.01 ± (0.09) Aa	11.76 ± (9.75) Ba	1.25 ± (3.33) Bb
Sussundenga	1 ± (2.56) Aa	0 ± (0.0) Aa	26.19 ± (24.73) Aa	0.74 ± (2.32) Bb
Vanduzi	0.44 ± (1.60) Aa	0 ± (0.0) Aa	10.56 ± (11.16) Ba	2.75 ± (5.59) Ab

SD = Standard Deviation. Means ± (SD) followed by the same capital letter in the column are not statistically different. Means ± (SD) followed by the same small letter between columns are not statistically different.

## Supplementary Materials

The following are available online at https://www.mdpi.com/2075-4450/11/8/512/s1, File S1: Analysis of Variance (percentage of damaged plants, percentage of infested plants, density of egg masses and larvae of the FAW) among districts within the same season; File S2: t-Test (percentage of damaged plants, percentage of infested plants, density of egg masses and larvae of the FAW) between seasons in the same district.
