# Supplementary material for: Seasonal Dynamics of the Alien Invasive Insect Pest Spodoptera frugiperda Smith (Lepidoptera: Noctuidae) in Manica Province, Central Mozambique
Source: Insects. 2020 Aug 7;11(8):512. doi: 10.3390/insects11080512 (PMC7469179; doi:10.3390/insects11080512)
Supplement: Supplementary file 1 [file insects-11-00512-s001.pdf]

Supplementary 1: Analysis of Variance among districts per season

ANOVA DRY SEASON

```
##Anova dry season
>
> DrySeason=read.csv(file.choose())
> attach(DrySeason)
> Damage=aov(Damage~District)
> Damage
Call:
   aov(formula = Damage ~ District)

Terms:
                District Residuals
Sum of Squares    6759.15 153204.43
Deg. of Freedom         3       136

Residual standard error: 33.56342
Estimated effects may be unbalanced
> summary(Damage)
            Df Sum Sq Mean Sq F value Pr(>F)
District     3   6759    2253       2  0.117
Residuals   136 153204    1126
>
```

```
> ##ANOVA INFESTATION
>
> Infestation=aov(Infestation~District)
> Infestation
Call:
   aov(formula = Infestation ~ District)

Terms:
                District Residuals
Sum of Squares   17778.43 194513.71
Deg. of Freedom         3       136

Residual standard error: 37.81862
Estimated effects may be unbalanced
> summary(Infestation)
             Df Sum Sq Mean Sq F value Pr(>F)
District      3  17778    5926   4.143 0.0076 **
Residuals   136 194514    1430
---
Signif. codes:  0 ‘***’ 0.001 ‘**’ 0.01 ‘*’ 0.05 ‘.’ 0.1 ‘ ’ 1
> TukeyHSD(Infestation)
  Tukey multiple comparisons of means
    95% family-wise confidence level

Fit: aov(formula = Infestation ~ District)
```

```
$District

                    diff       lwr       upr     p adj

Manica-Macate       17.448276  -9.398199 44.2947512 0.3326541

Sussundega-Macate   35.481481   8.178556 62.7844070 0.0051739

Vanduzi-Macate      11.627119 -11.847742 35.1019797 0.5719337

Sussundega-Manica   18.033206  -8.273867 44.3402787 0.2859543

Vanduzi-Manica      -5.821157 -28.129933 16.4876181 0.9050186

Vanduzi-Sussundega -23.854363 -46.710390 -0.9983361 0.0371804


> ##ANOVA EGGS

>

> Eggs=aov(Eggs~District)

> Eggs

Call:

   aov(formula = Eggs ~ District)


Terms:

             District Residuals

Sum of Squares   10.5407  402.1093

Deg. of Freedom       3       136


Residual standard error: 1.719502

Estimated effects may be unbalanced

> summary(Eggs)
```

```
            Df Sum Sq Mean Sq F value Pr(>F)
District      3   10.5   3.514   1.188  0.317
Residuals   136  402.1   2.957
>
> ##ANOVA LARVAE
>
> Larvae=aov(Larvae~District)
> Larvae
Call:
   aov(formula = Larvae ~ District)

Terms:
                District Residuals
Sum of Squares   5718.083 28361.767
Deg. of Freedom        3       136

Residual standard error: 14.441
Estimated effects may be unbalanced
> summary(Larvae)
            Df Sum Sq Mean Sq F value   Pr(>F)
District      3   5718  1906.0    9.14 1.49e-05 ***
Residuals   136  28362   208.5
---
Signif. codes:  0 ‘***’ 0.001 ‘**’ 0.01 ‘*’ 0.05 ‘.’ 0.1 ‘ ’ 1
> TukeyHSD(Larvae)
```

```
  Tukey multiple comparisons of means
    95% family-wise confidence level

Fit: aov(formula = Larvae ~ District)

$District
                          diff        lwr       upr     p adj
Manica-Macate         3.838621  -6.412676 14.089917 0.7644751
Sussundega-Macate    18.265185   7.839593 28.690777 0.0000670
Vanduzi-Macate        2.639322  -6.324528 11.603172 0.8696699
Sussundega-Manica    14.426564   4.381238 24.471891 0.0015501
Vanduzi-Manica       -1.199299  -9.717880  7.319282 0.9831484
Vanduzi-Sussundega -15.625863 -24.353412 -6.898315 0.0000444
```

ANOVA RAINY SEASON

```
###anova rainy season
>
> RainySeason=read.csv(file.choose())
> attach(RainySeason)
> Damage=aov(Damage~District)
> Damage
Call:
   aov(formula = Damage ~ District)
```

```
Terms:
                District Residuals
Sum of Squares   16389.3  584827.4
Deg. of Freedom        3       477

Residual standard error: 35.01504
Estimated effects may be unbalanced
> summary(Damage)
             Df Sum Sq Mean Sq F value  Pr(>F)
District      3  16389    5463   4.456 0.00423 **
Residuals   477 584827    1226
---
Signif. codes:  0 ‘***’ 0.001 ‘**’ 0.01 ‘*’ 0.05 ‘.’ 0.1 ‘ ’ 1
> TukeyHSD(Damage)
  Tukey multiple comparisons of means
    95% family-wise confidence level

Fit: aov(formula = Damage ~ District)

$District
                         diff        lwr       upr     p adj
Manica-Macate       -0.7330152 -11.786005 10.319975 0.9982217
Sussundenga-Macate  -7.4667212 -18.443111  3.509668 0.2971864
Vanduzi-Macate      10.9278188  -2.275132 24.130770 0.1439053
Sussundenga-Manica  -6.7337061 -17.563212  4.095800 0.3778423
```

```
Vanduzi-Manica      11.6608339  -1.420259 24.741927 0.0998652

Vanduzi-Sussundenga 18.3945400   5.378107 31.410973 0.0016945


> ##ANOVA INFESTATION

>

> Infestation=aov(Infestation~District)

> Infestation

Call:

   aov(formula = Infestation ~ District)


Terms:

                District Residuals

Sum of Squares   4795.70  79776.76

Deg. of Freedom        3       477


Residual standard error: 12.9324

Estimated effects may be unbalanced

> summary(Infestation)

             Df Sum Sq Mean Sq F value   Pr(>F)

District      3   4796  1598.6   9.558 3.86e-06 ***

Residuals   477  79777   167.2

---

Signif. codes:  0 '***' 0.001 '**' 0.01 '*' 0.05 '.' 0.1 ' ' 1

> TukeyHSD(Infestation)

  Tukey multiple comparisons of means
```

```
   95% family-wise confidence level

Fit: aov(formula = Infestation ~ District)

$District
                         diff       lwr       upr     p adj
Manica-Macate        3.0050533 -1.077239  7.087346 0.2303315
Sussundenga-Macate   0.6115657 -3.442435  4.665567 0.9800043
Vanduzi-Macate       9.3709168  4.494561 14.247272 0.0000060
Sussundenga-Manica  -2.3934876 -6.393239  1.606264 0.4127502
Vanduzi-Manica       6.3658634  1.534515 11.197212 0.0041013
Vanduzi-Sussundenga  8.7593510  3.951884 13.566818 0.0000205

> ##ANOVA EGGS
>
> Eggs=aov(Eggs~District)
> Eggs
Call:
   aov(formula = Eggs ~ District)

Terms:
                District Residuals
Sum of Squares  0.078401 16.869624
Deg. of Freedom        3       477
```

```
Residual standard error: 0.1880587
Estimated effects may be unbalanced
> summary(Eggs)
             Df Sum Sq Mean Sq F value Pr(>F)
District      3  0.078 0.02613   0.739  0.529
Residuals   477 16.870 0.03537
>
> ###ANOVA LARVAE
>
> Larvae=aov(Larvae~District)
> Larvae
Call:
   aov(formula = Larvae ~ District)

Terms:
                District Residuals
Sum of Squares   265.125  4766.884
Deg. of Freedom        3       477

Residual standard error: 3.161245
Estimated effects may be unbalanced
> summary(Larvae)
             Df Sum Sq Mean Sq F value   Pr(>F)
District      3    265   88.37   8.843 1.03e-05 ***
Residuals   477   4767    9.99
```

```
---
Signif. codes:  0 ‘***’ 0.001 ‘**’ 0.01 ‘*’ 0.05 ‘.’ 0.1 ‘ ’ 1
> TukeyHSD(Larvae)
  Tukey multiple comparisons of means
    95% family-wise confidence level

Fit: aov(formula = Larvae ~ District)

$District
                          diff        lwr      upr     p adj
Manica-Macate        0.7327906 -0.2651006 1.730682 0.2322973
Sussundenga-Macate   0.2222040 -0.7687715 1.213180 0.9386309
Vanduzi-Macate       2.2380400  1.0460450 3.430035 0.0000104
Sussundenga-Manica  -0.5105865 -1.4883011 0.467128 0.5338308
Vanduzi-Manica       1.5052495  0.3242561 2.686243 0.0059908
Vanduzi-Sussundenga  2.0158360  0.8406803 3.190992 0.0000713

>
```

Supplementary 2: t-Test of variables between season per district

DISTRICT OF MACATE

```
> ## t test percentage of damaged plants in Macate per season
>
> MacateDamage=read.csv(file.choose())
> attach(MacateDamage)
> t.test(DrySeason,RainySeason)

        Welch Two Sample t-test

data:  DrySeason and RainySeason
t = 4.9558, df = 33.086, p-value = 2.088e-05
alternative hypothesis: true difference in means is not equal to 0
95 percent confidence interval:
 25.38048 60.72721
sample estimates:
mean of x mean of y
 62.40000  19.34615

> ## t test percentage of infested plants in Macate per season
>
> MacateInfestation=read.csv(file.choose())
> attach(MacateInfestation)
> t.test(DrySeason,RainySeason)
```

```
	Welch Two Sample t-test

data:  DrySeason and RainySeason
t = 3.6329, df = 24.301, p-value = 0.001305
alternative hypothesis: true difference in means is not equal to 0
95 percent confidence interval:
 12.26954 44.49969
sample estimates:
mean of x mean of y 
31.000000  2.615385 

> ### t test Eggs Macate per season
> 
> MacateEggs=read.csv(file.choose())
> attach(MacateEggs)
> t.test(DrySeason,RainySeason)

	Welch Two Sample t-test

data:  DrySeason and RainySeason
t = 1.0046, df = 26.983, p-value = 0.324
alternative hypothesis: true difference in means is not equal to 0
95 percent confidence interval:
 -0.1347125  0.3931740
```

```
sample estimates:
 mean of x  mean of y
0.16000000 0.03076923

> ### t test Larvae Macate per season
>
> MacateLarvae=read.csv(file.choose())
> attach(MacateLarvae)
> t.test(DrySeason,RainySeason)

        Welch Two Sample t-test

data:  DrySeason and RainySeason
t = 3.5674, df = 24.17, p-value = 0.001547
alternative hypothesis: true difference in means is not equal to 0
95 percent confidence interval:
  3.122318 11.686913
sample estimates:
mean of x mean of y
7.9200000 0.5153846
```

DISTRICT OF MANICA

```
### t test percentage of damaged plantas in Manica per season
>
> ManicaDamage=read.csv(file.choose())
```

```
> attach(ManicaDamage)
> t.test(DrySeason,RainySeason)

	Welch Two Sample t-test

data:  DrySeason and RainySeason
t = 8.3841, df = 39.057, p-value = 2.897e-10
alternative hypothesis: true difference in means is not equal to 0
95 percent confidence interval:
 45.92371 75.12587
sample estimates:
mean of x mean of y
 79.13793  18.61314

> ## t test percentage of infested plants in Manica per season
>
> ManicaInfestation=read.csv(file.choose())
> attach(ManicaInfestation)
> t.test(DrySeason,RainySeason)

	Welch Two Sample t-test

data:  DrySeason and RainySeason
t = 6.4099, df = 30.02, p-value = 4.448e-07
alternative hypothesis: true difference in means is not equal to 0
```

```
95 percent confidence interval:
 29.18263 56.47304
sample estimates:
mean of x mean of y
48.448276  5.620438

> ## t test Eggs in Manica per season
>
> ManicaEggs=read.csv(file.choose())
> attach(ManicaEggs)
> t.test(DrySeason,RainySeason)

        Welch Two Sample t-test

data:  DrySeason and RainySeason
t = 2.2565, df = 28.033, p-value = 0.03202
alternative hypothesis: true difference in means is not equal to 0
95 percent confidence interval:
 0.06296593 1.30174587
sample estimates:
 mean of x  mean of y
0.68965517 0.00729927

> ### t test Larvae in Manica per season
>
```

```
> ManicaLarvae=read.csv(file.choose())
> attach(ManicaLarvae)
> t.test(DrySeason,RainySeason)

        Welch Two Sample t-test

data:  DrySeason and RainySeason
t = 5.7332, df = 29.395, p-value = 3.164e-06
alternative hypothesis: true difference in means is not equal to 0
95 percent confidence interval:
  6.763189 14.257702
sample estimates:
mean of x mean of y
11.758621  1.248175
```

DISTRICT OF SUSSUNDENGA

```
### t test percentage of damaged plants in Sussundenga per season
>
> SussDamage=read.csv(file.choose())
> attach(SussDamage)
> t.test(DrySeason,RainySeason)

        Welch Two Sample t-test

data:  DrySeason and RainySeason
```

```
t = 10.734, df = 34.694, p-value = 1.434e-12
alternative hypothesis: true difference in means is not equal to 0
95 percent confidence interval:
 56.43465 82.76945
sample estimates:
mean of x mean of y
 81.48148  11.87943


> ## t test percentage of infested plants in Sussundenga per season
>
> SussInfestation=read.csv(file.choose())
> attach(SussInfestation)
> t.test(DrySeason,RainySeason)


        Welch Two Sample t-test


data:  DrySeason and RainySeason
t = 8.6084, df = 26.646, p-value = 3.556e-09
alternative hypothesis: true difference in means is not equal to 0
95 percent confidence interval:
 48.16827 78.34080
sample estimates:
mean of x mean of y
 66.48148   3.22695
```

```
> ## t test Eggs in Sussundenga per season
>
> SussEggs=read.csv(file.choose())
> attach(SussEggs)
> t.test(DrySeason,RainySeason)

        Welch Two Sample t-test

data:  DrySeason and RainySeason
t = 2.0321, df = 26, p-value = 0.05248
alternative hypothesis: true difference in means is not equal to 0
95 percent confidence interval:
 -0.01153195  2.01153195
sample estimates:
mean of x mean of y
        1         0

> ## t test Larvae in Sussundenga per season
>
> SussLarvae=read.csv(file.choose())
> attach(SussLarvae)
> t.test(DrySeason,RainySeason)

        Welch Two Sample t-test
```

data: DrySeason and RainySeason

t = 5.3433, df = 26.087, p-value = 1.347e-05

alternative hypothesis: true difference in means is not equal to 0

95 percent confidence interval:

 15.6596 35.2356

sample estimates:

 mean of x mean of y

26.1851852 0.7375887

DISTRICT OF VANDUZI

PERCENT OF DAMAGED PLANTS

VanduziDamage=read.csv(file.choose())

> attach(VanduziDamage)

> t.test(DrySeason,RainySeason)

        Welch Two Sample t-test

data: DrySeason and RainySeason

t = 7.9352, df = 128.32, p-value = 9.054e-13

alternative hypothesis: true difference in means is not equal to 0

95 percent confidence interval:

 37.77231 62.86618

sample estimates:

mean of x mean of y

 80.59322 30.27397

```
> #EGG DENSITY
> VanduziEggs=read.csv(file.choose())
> attach(VanduziEggs)
> t.test(DrySeason,RainySeason)

	Welch Two Sample t-test

data:  DrySeason and RainySeason
t = 2.1151, df = 58, p-value = 0.03873
alternative hypothesis: true difference in means is not equal to 0
95 percent confidence interval:
 0.02362813 0.85772781
sample estimates:
mean of x mean of y
 0.440678  0.000000

> #INFESTATION
> VanduziInfestation=read.csv(file.choose())
> attach(VanduziInfestation)
> t.test(DrySeason,RainySeason)

	Welch Two Sample t-test

data:  DrySeason and RainySeason
```

```
t = 5.4951, df = 85.434, p-value = 3.968e-07
alternative hypothesis: true difference in means is not equal to 0
95 percent confidence interval:
 19.55506 41.72657
sample estimates:
mean of x mean of y
 42.62712  11.98630

> #LARVAE DENSITY
> VanduziLarvae=read.csv(file.choose())
> attach(VanduziLarvae)
> t.test(DrySeason,RainySeason)

        Welch Two Sample t-test

data:  DrySeason and RainySeason
t = 4.8973, df = 81.245, p-value = 4.866e-06
alternative hypothesis: true difference in means is not equal to 0
95 percent confidence interval:
  4.634661 10.977133
sample estimates:
mean of x mean of y
10.559322  2.753425

>
```
